# Supplementary material for: Structural Analysis of the ESCRT-III Regulator Lethal(2) Giant Discs/Coiled-Coil and C2 Domain-Containing Protein 1 (Lgd/CC2D1)
Source: Cells. 2024 Jul 10;13(14):1174. doi: 10.3390/cells13141174 (PMC11275157; doi:10.3390/cells13141174)
Supplement: Supplementary file 1 [file cells-13-01174-s001.zip › cells-3065473-supplementary.pdf]

Supplementary Materials:

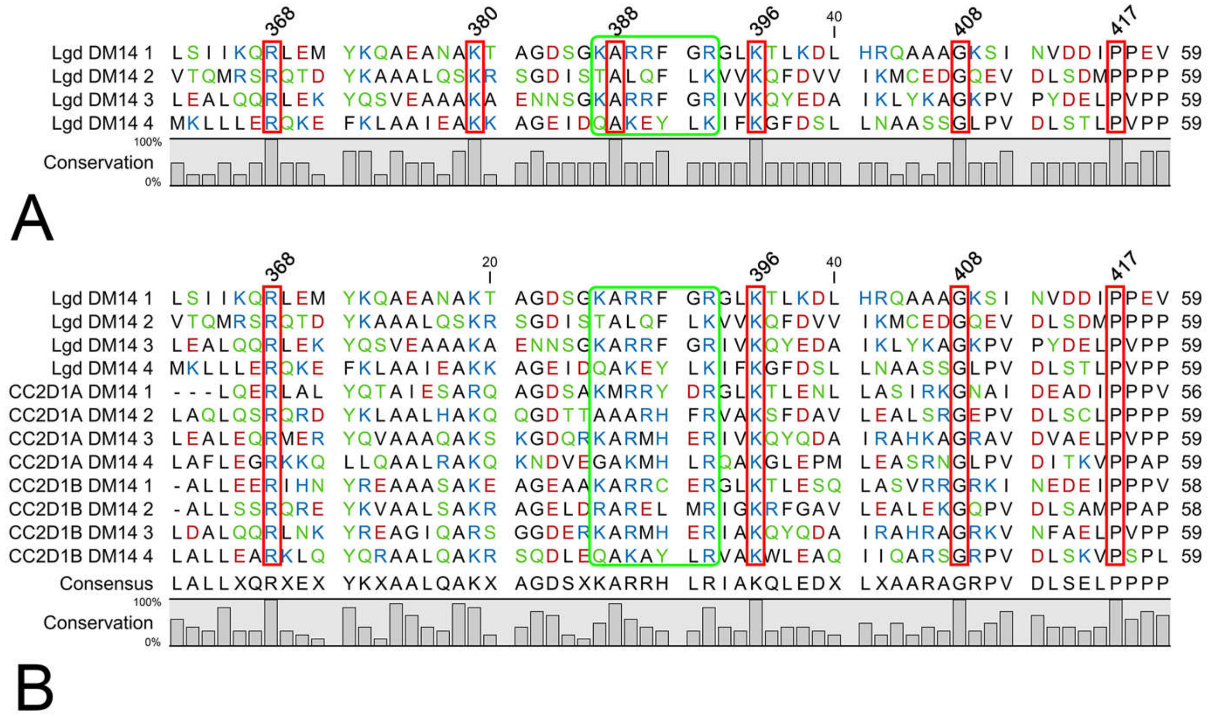

Figure S1. Sequence comparison of the DM14 domains. The green box highlights the regions of the KARRxxR domain in DM14-1 and DM14-3. (A) Sequence comparison of the four DM14 do-mains of Lgd reveals strongly conserved AAs at various positions (red boxes). (B) At four position the AAs are also conserved also among the mamalian Lgd orthologs (red boxes).

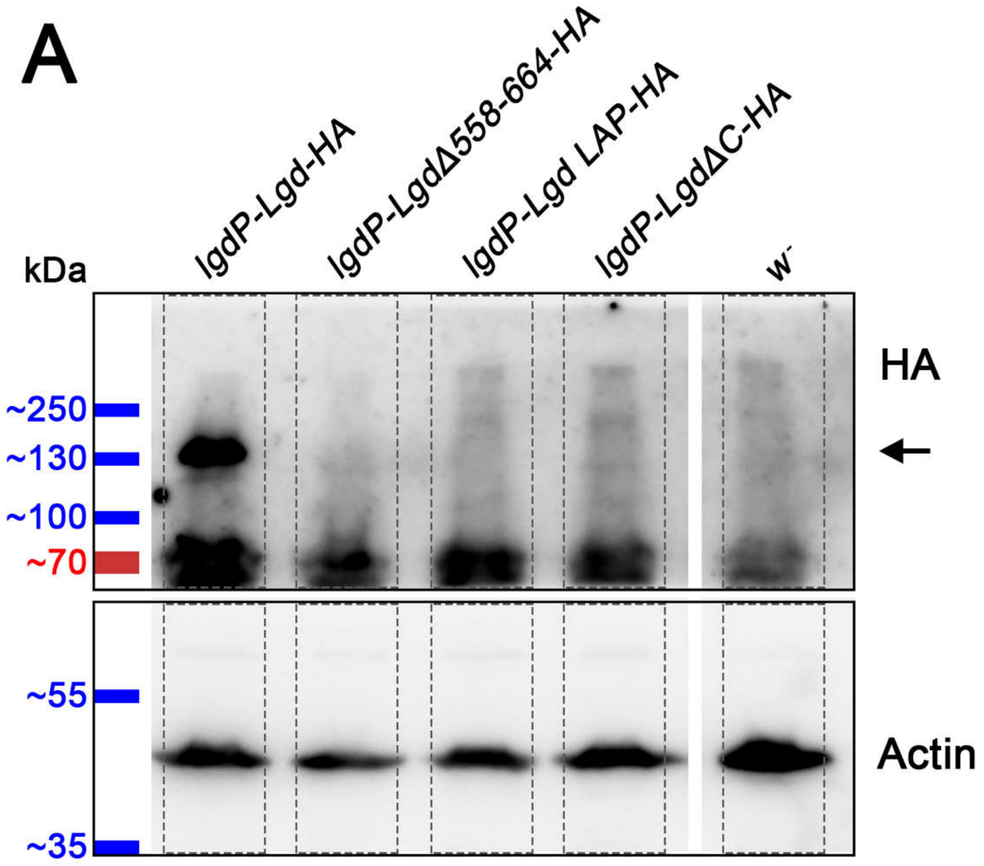

Figure S2. (A) Western-Blot of protein extracts of flies bearing the variants, revealing that the Lgd-variants with changes in the regions flanking the C2 domain are not expressed. The arrow point to the region of the expected Lgd signal.

Table S1. List of Primers used for Gibson cloning.

Primer-Name: Primer-Sequence 5′→ 3′
lgd N-term pattB For: taaacagcctctgtgcaatcaagg
lgd C-term pattB Rev: ttttatttaaattttcttgtttagccggtacc
lgd DM14 3,3 N-Term Rev: gctgcagagcctccaattgttgctcggcaatattattcttctg
lgd DM14 3,3 3(2) For: tattgccgagcaacaattggaggctctgcagcaacg
lgd DM14 3,3 3(2) Rev: gattatccctttgtgatggtggcacaggcagctcatc
lgd DM14 3,3 C-Term For: gctgcctgtgccaccatcacaaagggataatctagaagcc
lgd Del DM 14 2 Rev: attcaagaaactcagcaagaggattagggggagatgtgg
lgd Del DM 14 2 For: tccccctaatcctcttgctgagtttcttgaattccttaaaaaaatgc
lgd Del DM 14 4 Rev: gattatccctttgtgattgttgctcggcaatattattcttctgc
lgd Del DM 14 4 For: tattgccgagcaacaatcacaaagggataatctagaagcctc
lgd Del DM14 1 Rev: caattggctttacacttgtgtccacagtcgtcgtgg
lgd Del DM14 1 For: gacgactgtggacacaagtgtaaagccaattggcggg
lgd Del DM14 3 Rev: gcagcggtccaaaacccatatttgttgcagctgctagaac
lgd Del DM14 3 For: agctgcaacaaatatgggttttggaccgctgcctacg
lgd Del 1+3 (Del 3-4) For: agctgcaacaaatatgatgaaactgctcctcgagcgc

lgd Del 1+3 (Del 3-4) Rev: cgaggagcagtttcatcatatttgttgcagctgctagaac

Subcloning of C.e.LGD-Primer
Name Sequenz 5′→ 3′
Not I N Term C.e. lgd: ataagaatgcggccgcatgaatttcaatgatattgataatcaaatg
Kpn I C Term C.e. lgd: aatacttaaggggtaccttaattatctaaaatgagccatttctg

Mutagenesis-Primer (SDM) Name Sequenz 5′→ 3′
R368E For: g ttg gag gct ctg cag caa gag tta gag aaa tat caa tct g
R368E_Rev: c aga ttg ata ttt ctc taa ctc ttg ctg cag agc ctc caa c
DM14 3 For R>A R 368 A: g ttg gag gct ctg cag caa gcc tta gag aaa tat caa tct gta g
DM14 3 Rev R>A: ctacagattgatatttctctaaggcttgctgcagagcctccaac
K380E For: ct gta gaa gcg gca gcc gag gca gaa aac aat agc
K380E_Rev: gct att gtt ttc tgc ctc ggc tgc cgc ttc tac ag
K380A For: caa tct gta gaa gcg gca gcc gcc gca gaa aac aat agc gg
K380A Rev: ccgctattgttttctgcggcggctgccgcttctacagattg
DM14 3 For 1.K>R K 380 R: ct gta gaa gcg gca gcc cgc gca gaa aac aat agc gg
DM14 3 Rev 1.K->R: ccgctattgttttctgcgcgggctgccgcttctacag
K387E For: gca gaa aac aat agc gga gag gca aga cgc ttt gga agg
K387E Rev: cct tcc aaa gcg tct tgc ctc tcc gct att gtt ttc tgc
K387A For: ca gaa aac aat agc gga gcc gca aga cgc ttt gga ag
K387A Rev: ct tcc aaa gcg tct tgc ggc tcc gct att gtt ttc tg
K387R For: ca gaa aac aat agc gga cgc gca aga cgc ttt gga ag
K387R Rev: ct tcc aaa gcg tct tgc gcg tcc gct att gtt ttc tg
DM14 3 For A>G A 388 G: gaa aac aat agc gga aaa ggc aga cgc ttt gga agg att g
DM14 3 Rev A>G: caatccttccaaagcgtctgcctttccgctattgttttc
R389E For: gaa aac aat agc gga aaa gca gag cgc ttt gga agg att gtg
R389E Rev: cac aat cct tcc aaa gcg ctc tgc ttt tcc gct att gtt ttc
R389A For: gaa aac aat agc gga aaa gca gcc cgc ttt gga agg att gtg
R389A Rev: cac aat cct tcc aaa gcg ggc tgc ttt tcc gct att gtt ttc
R389K For: c aat agc gga aaa gca aag cgc ttt gga agg att g
R389K Rev: c aat cct tcc aaa gcg ctt tgc ttt tcc gct att g
R390E For: c aat agc gga aaa gca aga gag ttt gga agg att gtg aag c
R390E Rev: g ctt cac aat cct tcc aaa ctc tct tgc ttt tcc gct att g
R390A For: c aat agc gga aaa gca aga gcc ttt gga agg att gtg aag c
R390A Rev: g ctt cac aat cct tcc aaa ggc tct tgc ttt tcc gct att g
R390K For: c aat agc gga aaa gca aga aag ttt gga agg att gtg aag c
R390K Rev: g ctt cac aat cct tcc aaa ctt tct tgc ttt tcc gct att g
R393E For: gc gga aaa gca aga cgc ttt gga gag att gtg aag caa tac gaa g
R393E_Rev: c ttc gta ttg ctt cac aat ctc tcc aaa gcg tct tgc ttt tcc gc
R393A For: gga aaa gca aga cgc ttt gga gcc att gtg aag caa tac gaa g

R393A Rev: c ttc gta ttg ctt cac aat ggc tcc aaa gcg tct tgc ttt tcc
R393K For: gga aaa gca aga cgc ttt gga aag att gtg aag caa tac gaa g
R393K Rev: c ttc gta ttg ctt cac aat ctt tcc aaa gcg tct tgc ttt tcc
K396E For: cgc ttt gga agg att gtg gag caa tac gaa gat gcc
K396E Rev: ggcatcttcgtattgctccacaatccttccaaagcg
DM14 3 For 2.K>A K 396 A: cgc ttt gga agg att gtg gcc caa tac gaa gat gcc ata aag
DM14 3 Rev 2.K>A : ctttatggcatcttcgtattgggccacaatccttccaaagcg
DM14 3 For 2.K>R K 396 R: cgc ttt gga agg att gtg cgc caa tac gaa gat gcc ata aag
DM14 3 Rev 2.K>R: ctttatggcatcttcgtattggcgcacaatccttccaaagcg
R393E, K396E auf R For: cgc ttt gga gag att gtg gag caa tac gaa gat gcc ata aag
R393E, K396E auf R Rev: ctttatggcatcttcgtattgctccacaatctctccaaagcg
R393A, K396A For: gga aaa gca aga cgc ttt gga gcc att gtg gcc caa tac gaa g
R393A, K396A_Rev: c ttc gta ttg ggc cac aat ggc tcc aaa gcg tct tgc ttt tcc
DM14 3 For G>A G 408 A: g ttg tac aag gca gcc aaa cca gtg cct tac g
DM14 3 Rev G>A: cgtaaggcactggtttggctgccttgtacaac
DM14 3 For P>A P 417 A: g cct tac gat gag ctg gcc gtg cca cca ggt ttt gg
DM 14 3 Rev P>A: ccaaaacctggtggcacggccagctcatcgtaaggc
V 418 T: For g cct tac gat gag ctg cct acc cca cca ggt ttt gg
V 418 T Rev: ccaaaacctggtggggtaggcagctcatcgtaaggc
P 417 A, V 418 T For: g cct tac gat gag ctg gcc acc cca cca ggt ttt gg
P 417 A, V 418 T Rev: ccaaaacctggtggggtggccagctcatcgtaaggc

Primers for SOE-PCR: LgdΔ558-664
F C1: CGAATTCGCGGCCGCATGTTCTCCAGAAAGAAGC
R G2: CAACAATTTCAAGCTCACTAGGCGGGACGGGAAGAGTGC
F G3: CACTCTTCCCGTCCCGCCTAGTGAGCTTGAAATTGTTG
R C4: CTCTAGAGGTACCTCAAGCGTAATCTGGCACATCGTATGGG-TAAG

Primers for LgdΔ1-135
F aa2-135: CTAGCGGCCGCATGACGACGACTGTGGACACATTAAG
C4: CTCTAGAGGTACCTCAAGCGTAATCTGGCACATCGTATGGG-TAAG

Gibson-Assembly-Primer T.B.
Gibson C2-pattB Rev: GCGTTCCTATTTTATTTAAATTTTCTT-GTTTAGCCGGTACCTCAAGCG TAATCTGG
Gibson DM14 1 558 Rev: CAATGGCAAAGGAGGCTTCTAGAT-TATCCCTTTGTGAGACTTCAGGT GGAATGTCATCC

Gibson DM14 1 DM14 1 Rev: GCTTGTACATTTCCAGGCGCTGTTTGA-
TAATGCTTAAAAGAGGATTA GGGGGAGATGTG
Gibson DM14 4 Helix Rev 2: GCTTCACAATCCTTCCAAAGCGTCTT-
GCTTTTCCGCTATTGTTCGCTT TTTTGGCCTCTATAGC
Gibson DM14-4 LAP REV: GGCGGCGCCCTGAAAATAAAGAT-
TCTCAGGCGGGACGGGAAGAGTGC
Gibson Helix 3 to C2 FW 2: GCTATAGAGGCCAAAAAAGCGAACAA-
TAGCGGAAAAGCAAGACGCTT TGGAAGGATTGTGAAGCAT-
ACGAAGATGCCATAAAGTTGTACAAGGC AGGATTGCCAGTGGAC-
CTCAGC
Gibson LAP C2 Rev: CCCCGGACCACAACAATTTCAA-
GCTCCGAACCCGAGGGACCGCTCGAC
Gibson DM14 1 558 C2 FW: CAAGTCCATTAACGTG-
GATGACATTCCACCTGAAGTCTCACAAAGGG ATAATCTAGAAGC
Gibson DM14 1 DM14 1 FW:
CTACATCTGTGGCTCCCACATCTCCCCCTAATCCTCTTTTAAGCATTA
TCAAACAGCG
Gibson DM14 4 LAP FW: GGACCTCAGCAC-
TCTTCCCGTCCCGCCTGAGAATCTTTATTTTCAGGG CGCCGCC
Gibson LAP C2 FW: GCCCCTGTCGTCGAGCGGTCCCTCGGGTTCG-
GAGCTTGAAATTGTTGT GGTCCGGGG
Gibson pattB-lgd FW: GTTAATAAACAGCCTCTGTG-
CAATCAAGGGCGGCCGCATGTTCTCCA GAAAGAAGCC
